# Supplementary material for: Are early childhood stunting and catch-up growth associated with school age cognition?—Evidence from an Indian birth cohort
Source: PLoS One. 2022 Mar 2;17(3):e0264010. doi: 10.1371/journal.pone.0264010 (PMC8890627; doi:10.1371/journal.pone.0264010)
Supplement: Attachment — Submitted filename: Reply to editor and reviewers final.docx [file pone.0264010.s002.docx]

To

The reviewers

PLOS One

Re: PONE-D-21-21418 - Are early childhood stunting and catch-up growth associated with school age cognition? – Evidence from an Indian birth cohort

Dear reviewers,

Thank you for reviewing our manuscript titled “**Are early childhood stunting and catch-up growth associated with school age cognition? – Evidence from an Indian birth cohort**”. Author’s responses are provided below

| Comments | Authors’ response | Page number; line number |
| --- | --- | --- |
| Academic Editor |  |  |
| 1. Additionally, regarding line 137-138, “These contradictory findings may be due to differing definitions and time periods studied [20],” although the authors discussed it in the discussion, you are requested to address in the introduction in terms of what your study add to the literature. | Thank you. Suggested change has been made. | 6; line 142-147 |
| 2. Also the potential to recover from undernutrition to neurocognitive development need to be addressed. | Refer to the response to comment no. 1 | 6; line 142-147 |
| 3. In Table 1, “Weight-for-age Z scores (HAZ)” needs to be changed to “Weight-for-age Z scores (WAZ)” | Thank you. Changed to WAZ | Table 1 |
| 4. Financial statement | Deleted from Title page section | Cover letter |
| 5. Competing interest | No competing interest  Deleted from Title page section | Cover letter |
| 6. Data | Minimal anonymized data set is uploaded | Supplementary file |
| 7. Ethics statement – Mention only in manuscript | Deleted from Title page section |  |
| Reviewer 1 |  |  |
| 8. The authors note that the definition of catch up is a point of dispute in the literature, but do not explain why they selected the one that they used. The one used is a rather weak definition - children can be defined as having caught up even when falling behind in terms of CM deficits. Nonetheless, it is a commonly used definition and its use could be justified, but it does need to be justified and not used without question. | Thank you. Suggested changes have been incorporated and corresponding references have been added. | 7, Lines 175-178 |
| 9. -Stunting does not cause cognitive impairment, impairment and stunting are both indicators of malnutrition and possibly of lack of stimulation (and possible the combination of the two). As a result, any association between them needs to be carefully interpreted. The authors do not adequately cover this topic, which hinders the subsequent discussion of the results. | Thank you. Suggested changes have been incorporated and corresponding references have been added. | 19, Lines 365-372 |
| 10. If the authors are going to make the case that their results imply that nutrition programmes are needed, then a fuller discussion of the adequacy of the controls in the regression is needed. As mentioned, the association with stunting may be a result of smaller children being protected by parents leading to under stimulation - this may not be resolved by a nutrition program. Similarly, smaller children may be held back from school entry as parents do not think they are ready. The association between stunting and cognitive performance may be a result of less exposure to schooling. | Thank you. Our analysis showed that apart from early childhood stunting, other factors such as poor socioeconomic status and lesser IQ of the mother, were predictors for poor cognitive performance at 9 years in children. Also, our study showed that recovery from stunting at later years in children, who were stunted at 2 years, also to be a factor for better cognitive performance at 9 years. Thus, we would summarize that cognition at 9 years is because of interplay of multiple factors during the childhood period, which is inclusive of nutrition, timing of catch up growth, socioeconomic status and caregiver’s IQ. Changes have been made in the conclusion. | 20, lines – 404-406 |
| 11.Do the authors think that catch up growth could indicate a recovery from earlier malnutrition leading to a similar catch up in cognition? Or that those who catch up avoid falling further behind, while those who remain stunted continue to fall behind? Their results could support either argument, but which argument is correct has important policy implications and this should be discussed. | Findings from our study support the evidence that children who catch up perform better in cognition tests at 9 years, when compared to children, who have been persistently stunted between 2 and 9 years. This has been discussed in the lines 350-371 | - |
| 11 The authors from time to time slip into causal language, this should be avoided. | Thank you and this has been addressed | - |
| 12. The discussion on the importance of the first 1,000 days is a little unclear and could be improved. It is the pace and foundational nature of development in that period which is critical and this does not come through enough. | Thank you and this has now been addressed. | 19, lines 364-371, &  20, 391-393 |
| 13. Why report weight if you don't use it? Just for context? | Yes, weight for age scores have been provided to give a complete picture of anthropometric measurements of children in the cohort. | - |
| 14. The top third based on WAMI can hardly be called high SES given the selection process. Would relatively high not be better? | Thank you. We have used this nomenclature of high and low, having 33^rd^ percentile as cut-off consistently in the previous papers published from the same cohort. Hence, we would like to retain the same nomenclature.  Mohan VR, Sharma S, Ramanujam K, Babji S, Koshy B, Bondu JD, John SM, Kang G. Effects of elevated blood lead levels in preschool children in urban Vellore. Indian Pediatr. 2014 Aug;51(8):621-5.  Koshy B, Srinivasan M, Zachariah SM, Karthikeyan AS, Roshan R, Bose A, Mohan VR, John S, Ramanujam K, Muliyil J, Kang G. Body iron and lead status in early childhood and its effects on development and cognition: a longitudinal study from urban Vellore. Public Health Nutr. 2020 Aug;23(11):1896-1906. | - |
| 15. When was the WAMI measure taken? What the implications of only using one time point? Did those who exhibited catch up also see an improvement in their SES which might explain the better cognitive scores? | WAMI measures were available for the MAL-ED study, until 5 years of age and there were no WAMI measurements, thereafter. In the current study, WAMI scores measured at 2 years was considered, since we were interested to see, if SES status post-infancy continued to have effect on cognition scores measured at later childhood.  Further, subgroup analysis showed that there is no significant improvement in SES status between the children who were stunted at 2 years and recovered at 5 years vs. those who were stunted both at 2 and 5 years. To substantiate this, we would provide following data. Of 95 children, who were stunted at 2 years, 37 (38.9%) recovered from stunting, with 58 (61.1%) of them, continued to be stunted at 5 years. 4/37 (10.8%) children who recovered from stunting had the improvement in SES from low to high by 5 years; compared to 5/58 (8.62%) who continued to be stunted at 5 years and had a similar improvement in SES status from low to high. | - |
| Reviewer 2 |  |  |
| 16. Table 4: on the PIQ, it is shown that stunting at 2 y, though with a catch-up at 5 y had 5.77 PIQ less than normal children, but the children who stunted at 2 & 5 who only caught-up at 9 had much better PIQ than normal, than children who were already catch-up since 2y and those persisted till 9 y. What could explain such a pattern? Please add discussion | Thank you. We are unable to substantiate this pattern of regression co-efficients obtained between stunting with/without catch-up in early childhood and PIQ measurements at 9 years. The probable reason would be smaller sample size within the subgroups, that could have limited meaningful inference of the data. Having a smaller sample size in this study has already been highlighted as one of the limitations within the Discussion section. | 20, line 394 |
| 17. . Lines 365-366 & reference 17: Did children who received intervention were during older childhood (e.g., early school-age) and hence, the damage on cognition could have occurred since early age, aside from possible short duration of intervention? Please consider. | Thank you. We have added this into the Discussion. | 19, Line 383-384 |
| 18. 23. Editorial errors: a. Line 116, Introduction: ‘…. (HAZ) <2 on WHO…’, should this read ‘…. (HAZ) <-2 SD…’? b. Line 351 ‘A unit increase in HAZ <2 Y…’, suggest to read, ‘A unit increase in HAZ at <2 y of age..’ | Thank you.  Corresponding changes made | a. Page 5; line 117 b. Page 18; line 361 |

Thanking you

Yours sincerely

Authors
