# Supplementary material for: Are early childhood stunting and catch-up growth associated with school age cognition?—Evidence from an Indian birth cohort
Source: PLoS One. 2022 Mar 2;17(3):e0264010. doi: 10.1371/journal.pone.0264010 (PMC8890627; doi:10.1371/journal.pone.0264010)
Supplement: Attachment — Submitted filename: Reply to editor and reviewers.docx [file pone.0264010.s003.docx]

To

The reviewers

PLOS One

Re: PONE-D-21-21418 - Are early childhood stunting and catch-up growth associated with school age cognition? – Evidence from an Indian birth cohort

Dear reviewers,

Thank you for reviewing our manuscript titled “**Are early childhood stunting and catch-up growth associated with school age cognition? – Evidence from an Indian birth cohort**”. Author’s responses are provided below

| Comments | Authors’ response | Page number; line number |
| --- | --- | --- |
| 1. The authors have not substantially engage with a number of key issues raised in the previous review. Most notably, they have not justified their definition of catch up growth other than to say that other people use it, so it must be okay. They do not even mention what the other definitions are and how these could explain the contradictory findings that they also seek to explain. | Thank you.  Different definitions have been included in the introduction – page 6, lines 126-128.  Justification for current approach is included in methodology – page 8, lines 163-166 | 6; 126-128  8; 163-166 |
| 2. They also do not engage with the possibility that their results are endogenous - i.e. growth and cognition both have common causes. This links to perhaps the most serious problem - the authors use causal language throughout. Growing taller will not make you smarter. Growing taller may indicate that your environment has improved and that this environment now better facilitates cognitive development. This mechanism is not explained or referenced when interpreting findings. The absence of this explanation (understanding?) may explain why the conclusions are over stated. It may also explain why the results from the literature discussed are also over interpreted as showing a causal link. | Thank you.  We completely agree with the lack of causalilty and have used the word ‘association’ throughout manuscript.  We are happy to edit/modify if this is not clear in the manuscript.  Another statement also has been added to highlight common and intersecting risks and causative pathways for both growth and cognition in early childhood. | 20; 360-362 |
| 3. Finally, the authors ignored the need to differentiate between catch up in cognition and a slowing of the rate at which children are falling behind. If children who remain stunted are falling behind at a faster rate than those who have 'caught up', then a significant result in their regression may not be indicating catch up in cognition in the latter group. This is a critical point - it speaks to the extent to which inputs in different life stages are or are not substitutable. | Thank you.  That is a valid observation and we agree with you.  We have added further information in Table 2 to show that children in all groups improved in HAZ between 2 and 9 years of age. | Table 2 |

Thanking you

Yours sincerely

Authors
