# Supplementary material for: Are early childhood stunting and catch-up growth associated with school age cognition?—Evidence from an Indian birth cohort
Source: PLoS One. 2022 Mar 2;17(3):e0264010. doi: 10.1371/journal.pone.0264010 (PMC8890627; doi:10.1371/journal.pone.0264010)
Supplement: Attachment — Submitted filename: Reply to editor and reviewers.docx [file pone.0264010.s004.docx]

To

The reviewers

PLOS One

Re: PONE-D-21-21418 - Are early childhood stunting and catch-up growth associated with school age cognition? – Evidence from an Indian birth cohort

Dear editor and reviewers,

Thank you for reviewing our manuscript titled “**Are early childhood stunting and catch-up growth associated with school age cognition? – Evidence from an Indian birth cohort**”. Author’s responses are provided below

| Comments | Authors’ response | Page number; line number  (Track version of the manuscript) |
| --- | --- | --- |
| Further, there are several miner things which the authors should correct.  Table 3 showed the scores among 4 categories of stunting and catch up status of children and the coefficient of the rest three group, compared to the “never stunted group” were shown in Table 4. However, the authors used causal languages as the reviewer #1 mentioned. | Thank you for the comment and the category ‘never stunted’ in table 2, 3 and 4 is replaced with ‘children who were never stunted’ | Table 2, 3 and 4 |
| Thus, the uses of “reduction” or “decline” are not appropriate. You may use “lower score” instead. Please revise throughout the manuscript. | Thank you and this correction has been made in the Abstract, Results and Conclusions section. | Page 4 and lines 83 and 88  Page 15 and lines 284, 287 and 291  Page 22 and lines 406 - 410 |
| Reg “Catch-up growth was associated with higher verbal intelligence as well total cognition, but not the performance component.” higher verbal intelligence as well as total cognition than what? you mean “never catch up group”? Please clarify the sentences | Thank you and this sentence has been rephrased. | Page 18 and lines 318-319 |
| The authors did not discuss why the second group (catch up at 5 years) are negatively associated with performance IQ. | Thank you and this change has been made in the Discussion | Page 21 and lines 392-395 |
| Make some efforts on it, and reduce the length of “good follow-up rate of your cohort study” and add after the limitation of your study” to highlight the significance of your study in the discussion. | Thank you and this change has been made in the Discussion | Page 21 and lines 402-403 |
| Lastly, put the acronym in parentheses after the full term, if you use the term the first time. For example, IQ in the abstract, SD in the introduction and ANOVA in the method section. | Thank you and these suggestions have been incorporated in the Abstract, Introduction and Methods. | Page 4 and line 84  Page 5 and line 103  Page 10 and lines 214-215 |
| There are still come remnants of causal language, such as 'reduction' and 'decline', but they are not serious and could easily be left | Thank you and this correction has been made throughout the manuscript. | Page 4 and lines 83 and 88  Page 15 and lines 284, 287 and 291  Page 20 and line 360 |
| The justification of the definition use is weak, it mentions only the advantages and fails to consider the limitations (of which there are many). But as mentioned, previously, the definition is often used and so I guess its unquestioned use here is acceptable. | Thank you and the justification for the use of definition based on HAZ scores in this study has been provided in Methods section. | Page 8 and lines 170 - 173 |
| The one remaining issue which I would like to see addressed relates to the failure to engage with the comment I made that there may be no catch up in cognition. The additions to the table to show that the z-score increased don't help and add little. The definitions of the categories already provided the outline of this information. Besides, the question was not about growth but cognition. Perhaps my explanations have not been clear, so I will try with an example.  Three people enter a race (Tom, Fred and Joann). At the half way mark, Tom and Fred are 50meters behind Joann. When Joann crosses the finish line, Fred is still 50meters behind, but Tom is now 100meters behind. Can you say that Fred caught up? They all moved forward and he stopped falling behind, but he did not catch up.  This is a critical point, because if that 50meters at half way is lost forever and all you can do post the half way mark is prevent someone falling further behind, interventions in the first half of the race are not substitutable. This does not mean that interventions in the second half to prevent further falling behind are not important, but there should not be any suggestion that we can wait to the second half and catch up all the way.  In my mind, the possibility that there was no catch up in cognition should be mentioned. And this should be carried through to the conclusions, i.e. that the consequences of early deprivation on cognition may not be reversible, but we can act to prevent children from falling further behind. | Thank you and we agree with this suggestion and corresponding changes have been made throughout in the manuscript. Use of ‘Catch up in cognition’ in children who recovered from early life stunting has been replaced as the attainment of higher cognition scores in these children compared to persistently stunted children in relevant sections. | Page 4 and lines 85-86, 89-91  Page 18 and lines 318-319  Page 21 and lines 385-389  Page 22 and line 408-410 |

Thanking you

Yours sincerely

Authors
